# The Clinical Generational Interview. An instrument for family assessment

**DOI:** 10.3389/fpsyg.2024.1361028

**Published:** 2024-05-31

**Authors:** Giancarlo Tamanza, Marialuisa Gennari

**Affiliations:** Department of Psychology, Catholic University of Sacred Heart, Milan, Italy

**Keywords:** couple interview, family relations evaluation, intergenerational assessment, clinical interview, qualitative tool

## Abstract

Interviews are the privileged tool for carrying out qualitative research and clinical assessments on family relationships. Nevertheless, there are limited examples of interviews in clinical and psychosocial literature that are explicitly aimed at the evaluation of relational-family constructs. This paper presents the essential characteristics of the Clinical Generational Interview (CGI): an original tool for investigating and evaluating family relationships, that aims to combine the complexity of the subject being studied with the systematic and rigorous approach. It was created according to the following criteria: a flexible qualitative approach, the production and relational reading of information, intersubjective measurability and control of the inferential/interpretative process, and clinical use. Although it is organized in a structured and well-defined form and provides a precise system for encoding information, it is not a test, nor an algorithm that can be used in a mechanically diagnostic sense; it is a very versatile psychological tool that can be used in two different areas: the first is related to clinical research on family and couple relationships, the second to relational assessments. The contribution illustrates the path of construction and elaboration of the instrument, considering first of all its theoretical foundations and the constructs derived from them and around which the set of items is organized. The criteria for coding and analyzing the information thus produced and the different possible areas of application are then described. Finally, the theoretical and methodological characteristics of the instrument are also considered in relation to the main interviews in the literature in order to highlight differential particularities.

## 1 Introduction

Interviews are the privileged tool for carrying out qualitative research and clinical assessments on family relationships. Nevertheless, there are limited examples of interviews in clinical and psychosocial literature that are explicitly aimed at the evaluation of relational-family constructs.

The contribution illustrates the Clinical Generational Interview (CGI): a tool for investigating and evaluating family relationships (Cigoli and Tamanza, 2009). It aims to combine the complexity of the topic studied (family relationships) with the systematicity and rigor of an intersubjective analysis procedure. It was created according to the following criteria: a flexible qualitative approach, the production and relational reading of information, intersubjective measurability and control of the inferential/interpretative process, and clinical use.

Although it is organized in a structured and well-defined form and provides a precise system for encoding information, it is not a test, nor an algorithm that can be used in a mechanically diagnostic sense; it is a very versatile psychological tool that can be used in two different areas: the first is related to clinical research on family and couple relationships; the second to relational-generational assessments.

The CGI is built around the macro-construct of family generativity (Cigoli and Scabini, 2006). This construct was chosen and conceptually defined starting from the clinical and psychosocial literature on family relationships, especially taking the acquisitions developed within the relational-symbolic model into account. Generativity is conceived as a “synthetic measurement” of the complexity of bonds and, more properly, of the quality of the exchange developed between generations. Its reference and source of information is the couple, and its implementation is divided into three dimensions (or axes) concerning the origins of each partner, the formation and development of the couple relationship and the passage and transmission to the next generation (the child(ren)).

Several years of work were required to build the CGI, and several clinical and psychosocial research teams were involved. Qualitatively discriminating items had to be chosen for each analytical dimension. Subsequently, the calculation system was constructed in order to obtain an analytical measurement method (for each dimension/axe) and a summary of the total family generativity considered. Over time, the instrument has been used to investigate different domains of family relationships, both with reference to different stages of the life cycle (Tamanza et al., 2016; Gennari and Tamanza, 2018; Tamanza et al., 2019) and considering different clinical intervention contexts LIKE…(Molgora et al., 2014; Ranieri et al., 2016; Gennari et al., 2018). The application of the CGI to these different objects and contexts has confirmed the usability of the instrument in its structure and sequence of items and, at the same time, its adaptability about how it is applied. This concerned, in particular, the possibility of administering the CGI in a single time session, but also in multiple sessions, as well as meeting with the couple jointly, but also with individual partners.

Antecedents to the CGI can be connected to theoretical references from the psychodynamic panorama (aspects from relational psychoanalysis, gestaltism and systemic relational therapy) which, as mentioned, were then taken and translated into key concepts and a research methodology falling under the Relational-Symbolic Model (Cigoli and Scabini, 2006). This paper presents the essential characteristics of the tool and the elements that distinguish it from other types of interviews, the logic that entails its use, as well as the criteria for analyzing and interpreting the information produced.

## 2 Comparison with other family interviews

Clinical research on family relationships has widely used qualitative methodologies, developing some interesting tools for observation and analysis of interactions. Much more limited, however, are the examples of instruments aimed at structured analysis of discursive productions that:

- are explicitly aimed at the evaluation of relational-family constructs and, even less, attributable to the issue of generativity;
- are organized in a structured way and provide for a specific system of analysis and information encoding.

In our exploration of the literature, we have identified six interesting tools from a conceptual and methodological point of view which are relevant for dissemination and use. Table 1 presents a summary of the various interviews considered according to the identification of constructs, the setting (or detection unit), the encoding system and the measurement system. As can clearly be observed, this is a rather varied panorama consisting of very different tools, even if they have certain similar aspects.

**Table 1:** Comparing family interviews.

	Construct	Setting	Encoding unit	Measurement system
Oral history interview (Buelhman and Gottman, 1996)	Multidimensional	Couple (observant)	Individual couple	Quantitative
Current relationship interview (Crowell and Owens, 2004)	Synthetic (*The attachment system*)	Individual	Individual	Quantitative and qualitative
Camberwell family interview (Vaughn and Leff, 1976)	Multidimensional and Synthetic (*expressed sensitivity*)	Individual	Individual	Quantitative
Darligton family interview (Wilkinson, 2000)	Multidimensional	Individual	Individual	Quantitative
Structured family interview (Watzlawick (1966))	Multiple	Multiple (individual, couple, family)	Multiple	Qualitative
Personal history interview (McAdams, 1997)	Multiple	Individual	Individual	Qualitative
Generational clinical interview	Multidimensional and synthetic (*Generativity*)	Couple (interacting)	Individual couple	Qualitative and quantitative combined

As for content, it should be noted that only two tools are created with synthetic constructs: the Current Relationship Interview (Crowell and Owens, 2004) and the Cumberwell Family Interview (Vaughn and Leff, 1976), even if only the former has a construct referring to a precise and consolidated theoretical framework. The other tools refer to a plurality of variables and dimensions which, although they make an overall reading of the themes investigated possible, are not attributable to a unitary construct, in some cases also referring to different theoretical assumptions.

There is also a high degree of variability in the measurement systems adopted in the various interviews, both in terms of the founding method (in some cases the use of quantitative systems and in others qualitative systems), and the different degree of rigor, systematicity and inference. On the contrary however, they are much more similar in terms of the “setting” and encoding unit of the information produced, which is however almost always of an individual type.

The Clinical Generational Interview differs from each of these tools in the specificity of the reference construct and its clearly relational nature, the setting to produce information, the consequent encoding unit which attributes a distinctly peculiar significance to the couple, and lastly the measurement system tethered to the semantic evaluation of the propositional content, which includes a complex articulation of quantitative and qualitative elements.

The Clinical Generational Interview is placed within a precise theoretical-methodological reference that identifies the distinctive and identity-constituting character of the family within generativity. It is a “three-dimensional macro-construct” (origins, couple, passage to the next generation) that identifies the crucial dynamic and evolutionary junction of its constitution in the couple. From a procedural point of view, it follows that the joint meeting with the couple becomes the elective setting to produce information. The interview must also be conducted using specific relational methods that take the following aspects into account:

- management of the exchange methods to facilitate the dialogue-conversational-imaginal production of both partners in relation to the proposed themes, and respectful of the specificities of the joint setting;
- different modulation in introducing questions and regulating communication. The first part of the Interview (related to the partners’ origins) is in fact addressed to each partner, always in the presence of the other, while the second and third parts (couple and passage) are jointly addressed to the couple. In fact, it is as if the first part is an interview “in couple” (the other partner is present and may speak) and the second an interview “of couple”;
- the interviewer must behave in a way which solicits openness from the parental couples and their active involvement in the task they have been proposed. Dialogue is exchanged both with the interviewer and between the partners themselves, and discursive production should be encouraged and facilitated, allowing it to develop according to the progress of the couple’s exchange itself, rather than according to a rigid and mechanical sequence of questions and answers.

In other words, the Interview should be conducted with the couple using the typical style and sensitivity of clinical work.

## 3 The path of construction and validation of the instrument

**Figure 1:**
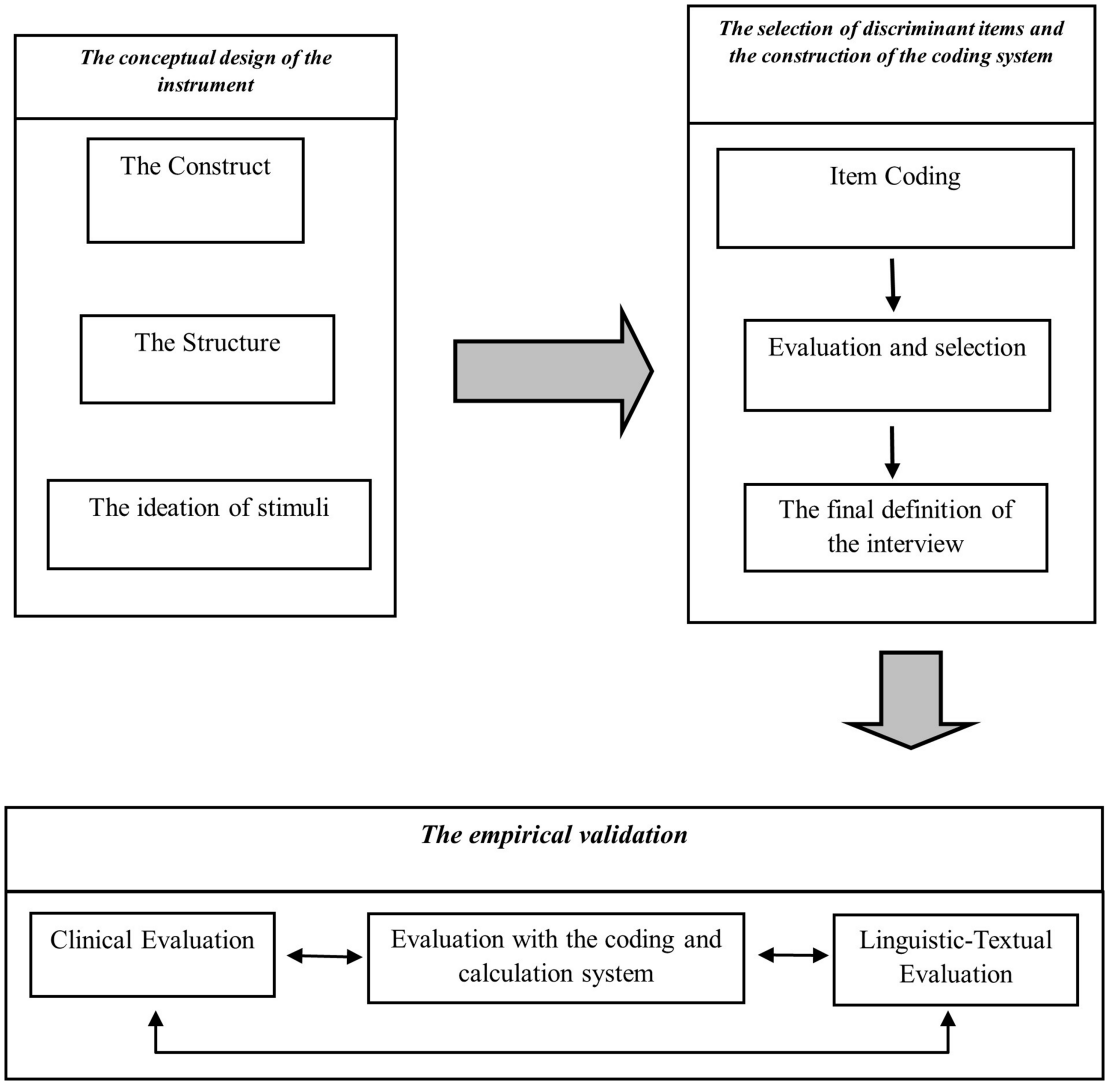
The path of construction and validation of the instrument.

The path of construction of the CGI was developed in three distinct and logically consequential stages (see Figure 1): the conceptual design of the instrument; the selection of the discriminant items and the construction of the coding system; the empirical validation of the instrument based on a normative sample.

The first phase of the work was carried out according to an *up-down* logic, that is, from a theoretical-conceptual vision assumed *a priori*. It consisted of two moments: the identification of the synthetic construct (generativity) and its tripartite articulation (i.e., the three analytic dimensions) and the identification of a set of verbal and imaginative stimuli. They were initially constructed by the research team during some “ideative production” sessions and then selected based on their comprehensibility and relevance through the contribution of some “focus groups” conducted with clinical psychologists and family psychotherapists. In this way, a “preliminary version” of the interview was obtained, which, in the second time, was administered to a sample of thirthy parents’ couples^1^ for the purpose of verifying its usability, i.e., to assess the discriminating value of each stimulus and thus obtain an effective and manageable version of the instrument.

This second phase of work involved four steps: the construction of the coding system for each stimulus, the identification of the computational rules for measuring each axis/size, the selection of the discriminating stimuli for each axis/size, and the definition of the final version of the interview. In this second phase, the path followed a *bottom-up* logic, like the process of “*item analysis*” typically used in the construction of metric scales^2^, with the relevant difference that in this case it involved the development of a system of analysis and measurement of *dialogic-discursive* material coded in categorical terms, including also the interrelation of three different analytical measures and the construction of a single summary assessment.

This second version of the interview, significantly smaller than the preliminary version^3^, was then administered, in the third phase of the course, to a second sample^4^ and the results thus obtained were the subject of two further analyses carried out quite independently: a *clinical evaluation* and a *linguistic-textual analysis*.^5^ This was done to be able to carry out a comparative evaluation of the results produced through the three different modes of analysis and thus to be able to obtain further elements of validation (or possible disconfirmation) of the adequacy and reliability of the CGI.

## 4 Thematic contents

In its entirety, the CGI consists of 23 openings for dialogue and two series of graphic-pictorial stimuli (paintings by authors), divided into three sections: the relationship for each partner with his/her origins (8), the couple relationship (9) the generational passage (6).

All the stimuli (dialogue-pictorial) were created by the research team during creative production sessions and subsequently chosen based on their comprehensibility and relevance, thanks also to the contribution of some “focus groups” conducted with clinical psychologists and family psychotherapists. The creation of stimuli was based on the following criteria:

- congruence with the conceptual construct (generativity) and its articulation in the three distinct dimensions/axes;
- production of representational elements and actions related to the affective and ethical dimensions of the family bonds (Cigoli and Scabini, 2006) concerning the three dimensions/axes.

These different stimuli were conceived in terms of integration, in the sense that the imaginative stimuli were introduced with the aim of promoting the partners’ reflection and verbalisation of their own family experience and couple dialogue, and not as independent indicators of the variables considered. This is why they are considered together with the discursive productions that accompany them in the subsequent encoding system. As will be better illustrated in the following paragraph, these stimuli were also the subject of preliminary work aimed at selecting the most suitable images for eliciting certain emotions, by grouping them into homogeneous categories.

First, we shall present the stimuli related to the first section (see Table 2).

**Table 2:** Relationship with the family of origin.

Item	Description
Warm-up	First of all, we ask you to immerse yourselves in your *origins*, i.e., the living environment, places, historical moment, traditions, family and extended family relationships as if you were going back in time and are seeing these things from your eyes as a child. Your mind will evoke images and scenarios. We want you to focus on them. We will give you both a few minutes to do this, closing your eyes if you like.
1.1	Good, now can you show us your living environment, each his or her own?
1.2	Thinking about your family, what were the important moments of family life? Go back in your mind to both everyday life and to particularly significant moments in family life. What was happening?
2	What were the “golden rules” of family life for relationships within the family and with the outside? From whom and how were they supported?
	Think of some childhood memories about:
3	Your relationship with your mother
4	Your relationship with your father
5	The relationship between siblings (if you did not have siblings, between cousins or friends)?
6	Now look at these images (reproductions of landscape paintings are shown). Silently, please each choose one that expresses and shows your environment of origin. Can you comment on the image you have chosen?
7	What did you learn in your family of origin about couple relationships and couple life? Were there “golden rules” on this subject too? Give me an example of the relationship your parents had through one or two memories.
8	Can you tell me, again with memories, about the relationship your parents had with their families of origin? What happened?

The first Interview section is related to origins. This dimension regards each member of the couple specifically and in a differentiated manner. The relative items are therefore addressed and referred to each partner and are encoded separately for each of them. However, it is conducted in the presence of both partners, who are also invited to comment on the choices and responses of the other at the end of their discussion.

After the presentation of the objectives, the interview begins with a moment for “warming-up.” This helps facilitate the couple’s involvement in the proposed task and helps them mentally place themselves in their own generational history. It then proceeds in alternation, asking one partner the questions first, and then the other partner.

The first Interview questions investigate the content and quality of the representations related to the origins. That is, they are aimed at exploring how the partners mentalize their family and cultural origins and the representations and affections that characterize them. The opening question/stimulus reveals various aspects:

1.1 Firstly the capacity/willingness of the couple to “go back in time.” The act of “letting go,” also by closing the eyes, conveys whether or not you trust in the clinical context, as well as the partners’ available mental resources. The producers of images and scenarios are therefore the partners themselves, and their focus is not on the confusion or the “void” of an image, but what actually results.

There are similarities here between the Interview and Gestalt’s techniques.

Last but not least, “showing the other” is a way to immediately put the relationship on the playing field. In doing so, one partner can interpret the other’s representations of his or her “origins,” gathering similarities as well as profound differences. Both a disqualification of others’ experiences and a lack of cognitive-sentimental focus correspond to deficits in the relational matrix.

The researcher-clinician also forms his or her own representation. Thus, in turn, he or she can intervene both to clarify and to further certain aspects. After this part of the interview, the attention is directed to family rituals (presence/absence and quality).

1.2 “Origins” and “rituals” are in fact connected to each other. The poor mentalization of contents and affects also includes the absence of significant rituals (ruinous typology); mentalization with open and suspended problems and widespread negativity also includes uncertain and confused rituals (critical typology); mentalization which is rich in contents and affections is associated with an active and heartfelt ritual (productive typology). Gregory Bateson (1972) discussed “heart algorithms” in this regard, meaning that family life practices, and in particular rituals, reveal meaning with regard to relational exchange. For our part, we attribute a sacred dimension to the origins (Cigoli, 2006), and a lack of sacredness has negative effects on family bonds.

Questions 2/5 seek to further, in specific terms, the re-evocation of the “environment of origin” previously carried out. There are two semantic areas solicited: the profound dimension of rules (the “golden rules”) of family life and the memory of relational events with the most significant figures, and the relative range of feelings inherent in the maternal-paternal-fraternal relationship.

At this point the first task concentrating on images is proposed, asking the couple to choose their “landscape of origin.” Thanks to their specific polysemic nature, the introduction of pictorial stimuli aims to activate imaginative thinking in the subjects (poiesis), and also aims to enrich dialogue. In fact, the entire interview is held in the presence of the partner, who is reciprocally invited to comment on the other’s choice.

**Figure 2:**
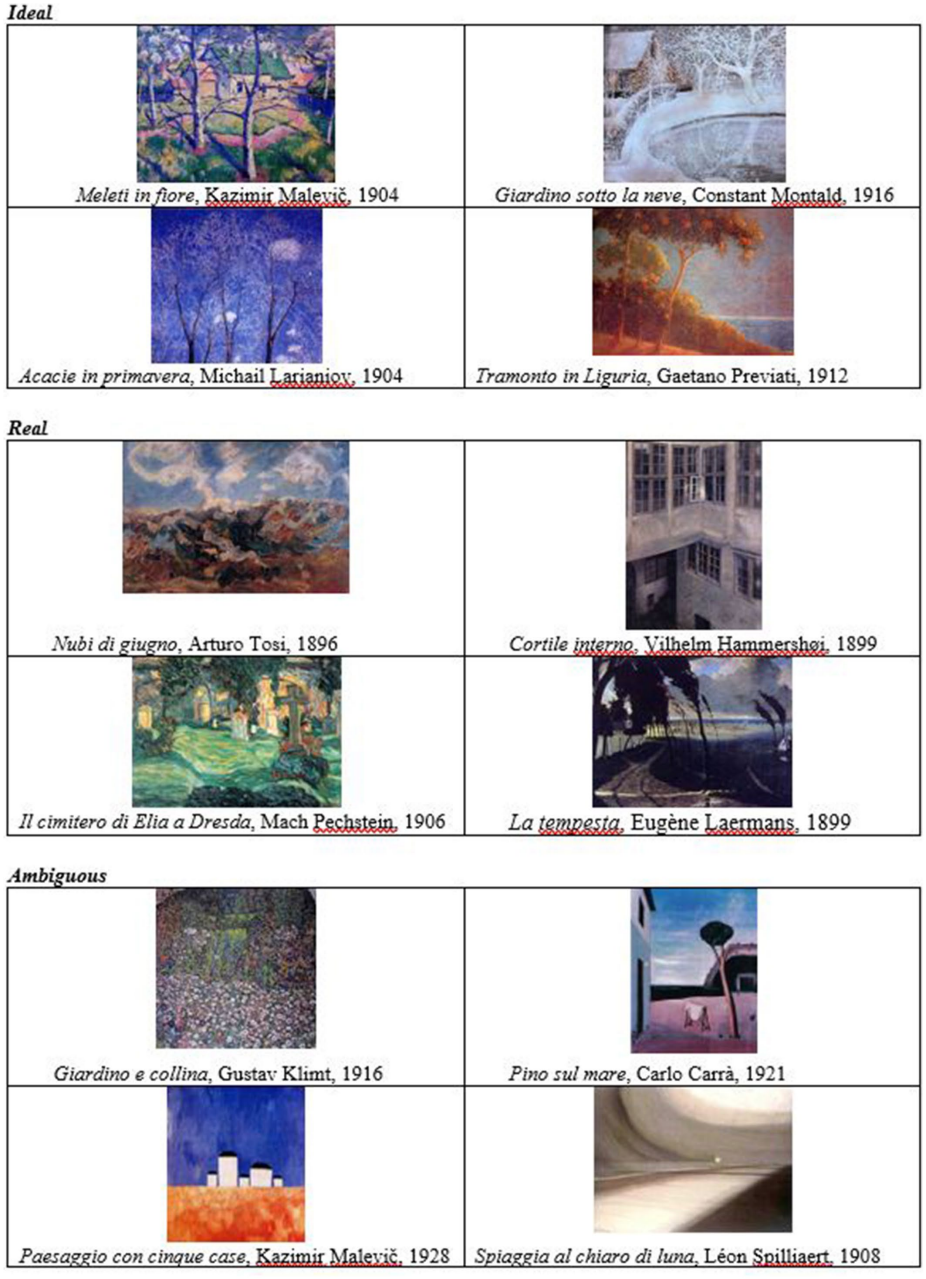
Landscape painting. Reproduced from Cigoli and Tamanza (2009, p. 117-120).

The images were chosen based on the identification of three categories highlighted by research work on landscapes in painting (Cigoli, 1999; Büttner, 2006). The categories of the images are: ideal landscapes, real landscapes and ambiguous landscapes (see Figure 2). The ideal landscapes include paintings where the artist’s intention is to communicate mental states of “paradise,” a “golden age,” an “ideal well-being.” The real landscapes include paintings where the artist’s intention is to communicate the “lived life,” “good and bad weather,” a “conflictual state” (heat and cold, light and shadow, the virginity of nature and the occupation of man, etc.). The ambiguous landscapes include paintings where the artist’s intention is of an uncertain nature, disturbing and alienating.

It is important to note that the categories of chosen images, both as regards the landscapes of origin and the couple scenarios, have nothing to do with the normal/abnormal, healthy/ill, correct/incorrect polarities and the like. Indeed, the images are to be considered visual stimuli that encourage a choice and not indicative in themselves of unique and discriminating meanings. What matters is how the partners react to and consider the image from a cognitive (perception, focus of attention, communication), affective (referring to the world of emotions-feelings) and ethical (referring to the value, or less, of the bond) point of view. Lastly, what matters is how the partners talk to each other. In this regard, the criteria related to communication (according to attention, socio-emotional and prognostic variables) that have been highlighted by systemic research for many years are valid.

The following questions (7/8) put the partners in “third-party position,” meaning they become observers involved in the relationships of others, whether they are those of a parenting couple of origin or those between parents and families of origin (in the sense of clan membership, ethnicity, lineage).

Striving for “having learned” (questions 7/8) serves to introduce, in a latent way, the identification issues affecting the generations. In the same way the problem-theme of the relationship between families of origin is introduced through the couple.

The following Table 3 presents the stimuli related to the second section: the couple relationship.

**Table 3:** The couple relationship.

Item	Description
	At this point the interviewer turns to both partners and lets them continue the dialogue.
1	How did you meet?
2	What made this meeting turn into a bond? (optional extension: Did you promise each other anything?)
3	What do you think you married in the other?
4	Did you find what you were looking for in each other?
5	What new aspects have you discovered in the other?
6	Have there been any particularly difficult moments in the relationship? And how did you deal with them? (was there forgiveness?)
7	Now you have a series of images before you (reproductions of paintings of couples are presented). Each of you should choose one to express how you experience your couple’s relationship, the feelings you have. Can you comment on the image you have chosen? Can you comment on the image chosen by the other?
8	Now I would like you to talk about the interaction between you as a couple and your families of origin, with her/his family. Could you explain the interaction with some episodes taken from everyday life, or even through metaphors or images?
9	How do you envision your future as a couple?

The second section of the interview concerns the couple relationship. The related questions are addressed and refer to the couple as a whole, and the partners themselves will decide how to respond and react to the questions asked. The encoding of the information produced is based on the response of the couple as such.

The interactive-communicative dynamic is not analytically encoded (microanalysis), but constitutes useful information about the adequacy of the interviewing process. In this regard, the researcher is advised to take note, on a separate sheet, of the recurring methods of exchange, as is traditional in interactive research (and clinical setting) on family relationships. It is also one of the fundamental elements the interviewer uses to modulate and manage the relationship with the couple. As mentioned, it is important for the couple to engage in the proposed dialogue according to its own methods, also ensuring that each partner expresses his or her position in relation to the proposed themes.

The first question (“How did you meet”) refers to Watzlawick’s (1966) famous Family Interview. It is however formulated as an opening based on dialogue, immediately followed by mentally urging the partners to grasp the difference between interaction and bond (question 2). Interactions are innumerable, but only few become a bond characterized by needs and desires that interlock and require a response. The crucial theme of the promise can also be introduced. Following Hanna Arendt (1958), we hold that this bond cannot hold true through life, living and its trials without any promises having been made. The Relational-Symbolic Model considers “secret interweaving” and the “promise” as the crucial dimensions of a couple bond (Cigoli and Scabini, 2006).

The following questions (3/5) imply a strong reference to the interpersonal plane (the relationship with the other) and projective and interjective identification processes. They are aimed at focusing the couple’s dialogue on the foundations of the bond, which in this case concern the previously mentioned “secret understanding,” i.e., the latent and often unconscious dimension of the bond itself. As can easily be understood, it is important to invite the partners to explore sensitive and very delicate topics and contents, and the interviewer must maintain a trusting and collaborative atmosphere, avoiding making the members of the couple feel excessively exposed or threatened in relation to any intimate and vulnerable aspects. In any case, the “climate” the interviewer senses is one of the indicators to be taken into account.

Question 6 instead focuses on the relationship-bond as such. It assumes that there is no bond without conflict (the soul of the relationship) and difficulties which must be faced, and calls on the couple’s commitment to cope with it. The “commitment” variable is very developed in the psychosocial research related to bonds and can be considered an analogue of the promise (to have an obligation, to pledge) that can be assumed, fragile, disqualified and attacked. “Promising,” however, is knowing how to “go beyond” the same perspective, while commitment concerns the resonance of the word given in the present. In any case, it is a “good accompaniment.”

Then a second task is proposed which is focused on images, this time with a new choice of pictures that concern “couple’s scenes.” The methods and aims of the proposal are substantially the same as those already illustrated in relation to the first series of images (the “landscapes of origin”): they favour the partners’ identification with the relationship by soliciting psychological characteristics. In short, the subject the images depict is not as important as the effect they have on an affective-ethical level.

**Figure 3:**
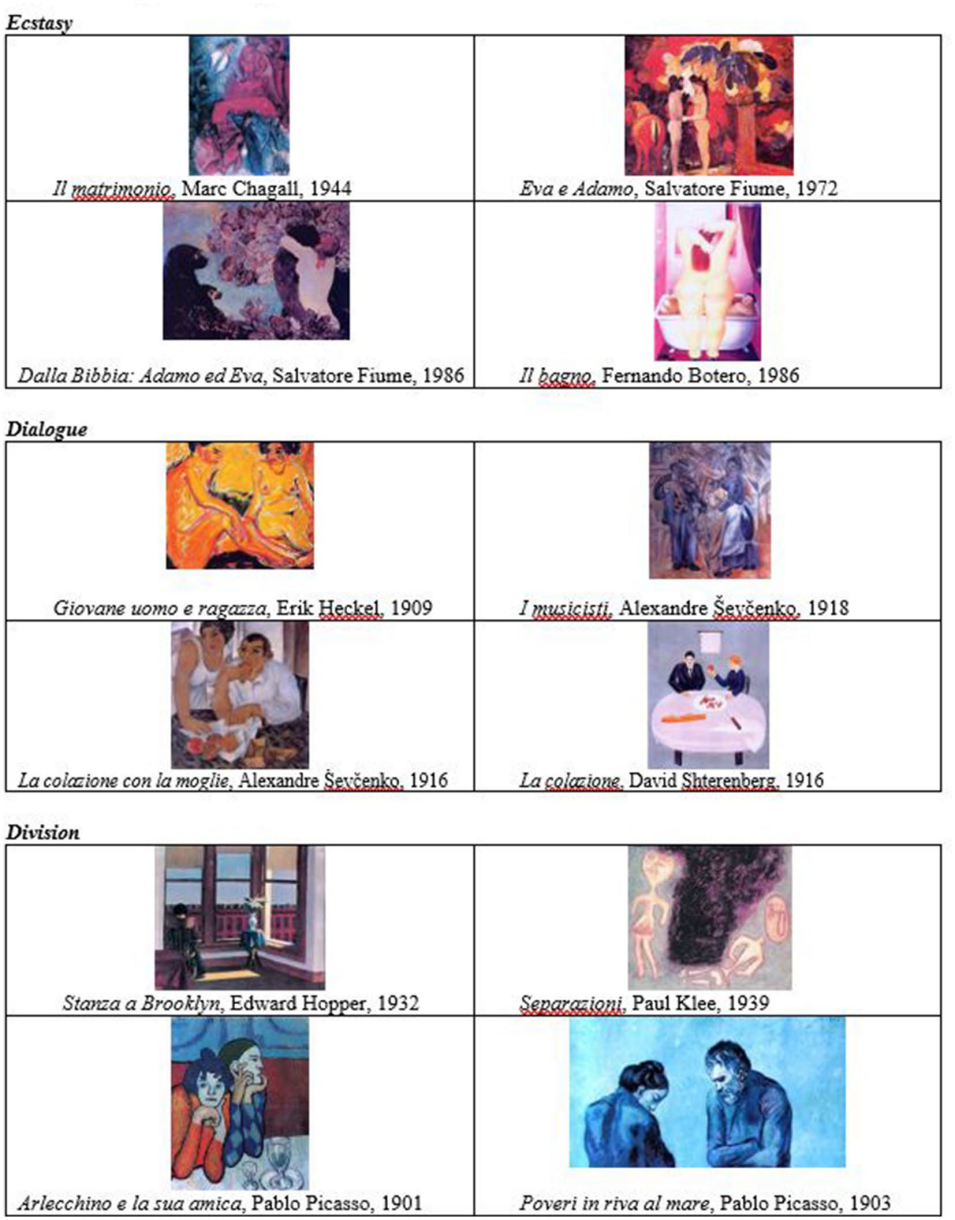
Couple’s painting. Reproduced from Cigoli and Tamanza (2009, p. 117-120).

In this case the three types, with four pictures for each of them, are the following: ecstasy, dialogue, division (see Figure 3). Ecstasy refers to paintings in which the artist’s intention is to communicate the presence of an ecstatic, fusional, paradisiacal, idyllic relationship: the couple transcends the everyday and “escapes” the present. Dialogue refers to paintings in which the artist’s intention is to compare the male and the female, to consider the similarities and differences and any shared aspects: the couple is at the forefront and takes each other by the hand. Division refers to paintings in which the artist’s intention is to highlight the presence of a fracture, isolation, discord: the couple is in a painful, broken, anguished, desperate state. After choosing the image, the partners are explicitly invited to comment on the choice made by the other, in order to enrich the couple’s dialogue and thus bring out the characteristics of the bond.

As the literature on family relationships has clearly highlighted, it is impossible to divide the couple relationship from that of both partners’ families of origin, in the sense that it is an integral part of the same bond. For this reason, the theme of the “other” (the other lineage) and the modalities of reception or rejection are introduced at this point in the interview. The partners’ experiences and above all the couple’s dialogue (the commentary) help us add another piece to the “puzzle” of the couple bond and its qualities, considering the relationship between the couple and their respective families of origin. In various cultures, whether patriarchal or matriarchal, cognatic or bilinear, the relationship between lineages and classes of belonging, as well as socio-economic status, involves and even anticipates the couple bond.

The “family of origin” should be understood as a place of interaction/clash, of domination and marginalization, of communion and rejection or isolation. In other words, the generative basis (and its drama) does not remotely end with the parent–child relationship, but opens up to the causes in a vertical sense and to the exchange between memberships and clans, also involving souls and the dead (Cigoli, 2006).

The last question (9) of this part of the Interview features an imaginative opening onto the future. It is obvious that the future being discussed, as well as the past, is in the present of the relationship, as taught by St. Augustine. This is how we have another source available for qualifying the “reality” of the couple bond. Furthermore, opening with the future helps us introduce and prepare the third part of the Interview relating to parenting (see Table 4).

**Table 4:** The generational change.

Item	Description
	The interviewer always turns to both parents-partners and lets them carry out the dialogue.
1	Before getting married, or becoming a couple, how did you imagine family life? Can you give some general examples?
2	In the reality of everyday life, which examples have come to be true, and which have not? What has been the same and what has been different?
3.1	What do you consider important to pass on to your children? What values, what life models?
3.2	Do these things relate to what your parents passed on to you?
4	Do you think you are able (or have been able) to pass on these values and life models? (What can be an obstacle? And a resource?)
5	Think of your children (in the case of more than one). Who do you think they have received this information from, and what is specific about them?
6	What has caused more pain and what has given hope/trust to family life?

The third part of the Interview concerns generational change. While the first two parts of the interview can be used in clinical and research contexts that involve all couple situations, this third part of the CGI explicitly refers to couples with children, i.e., families. Also in this case, the related questions are addressed and refer to the couple as a whole and the parents themselves decide how to respond and react to the proposed questions. The encoding of the information thus produced will result in a parental couple evaluation as such.

The thematic areas that are explored concern the prefigurative capacity (1) of the parent-partners (a method of “taking hold,” or less, on the future) and their ability to compare the examples with the actual family reality (2). “Equal” and “different” introduce the themes of coincidence and surprise that can be experienced positively or negatively. The next two questions (3.1 and 3.2), which due to their connected meaning are encoded together, aim to connect the world of values and life models with generational change and with the recognition that parents are themselves also children. Is there continuity and a transformation of values through the generations, or a break? The theme of lineage (“your parents”) is also proposed again.

Cognitivist-oriented family research appropriately insists on the aspect of parental effectiveness (4): an expectation is one thing, while the result of an action from which specific feelings arise is another, such as satisfaction or serious disappointment. Moreover, it is easy for the parents to involve the social scene, which can be considered as helpful or harmful.

Lastly, the parents’ dialogue regarding their children addresses the presence of both continuity and differentiation (5). A child is such only if he “has inherited,” but is also recognized for his specific traits. This concerns each child, and so it is not a matter of considering them “in equal parts,” but each according to their specificity (his “own”). This is the challenge.

The last question (6) is intended to recapitulate, as it invites people to reflect on aspects of family history that have spread grief and hope in relationships. Its purpose is to evaluate the ability of parents to recognize risky and resourceful elements inherent in the bonds. But here they are specified in terms of hope and trust which, not by chance and together with justice and equality, are recognized as the symbolic foundation of bonds. We will thus have, still considering the inherent fragility in the bonds between people, cases in which trust and hope are brought to safety and sustained and cases in which they collapse in deep distrust and despair. The Model that guides the Interview, taking up some old wisdom, underlines how the family climate is a decisive factor (not directly causal) in the construction of its members’ personalities. The result is that the so-called “quality of the relationship” is not measured in terms of satisfaction, communication, problem-solving, affective expression and so on, but precisely in terms of trust/mistrust, hope/despair, justice/injustice. We could even consider them from two different psychological languages.

## 5 The encoding and measurement system

The CGI uses a dual encoding system: typological and taxonomic (Bailey, 1994).

In our case, the taxonomic classification is made up of the set of “semantic categories” through which each textual/discursive unit is encoded and is therefore variable and specific for each item/question. This has also been built empirically (bottom-up) from the verbal productions present in the normative sample and is, by its nature, a classification which is open and can be integrated.

**Figure 4:**
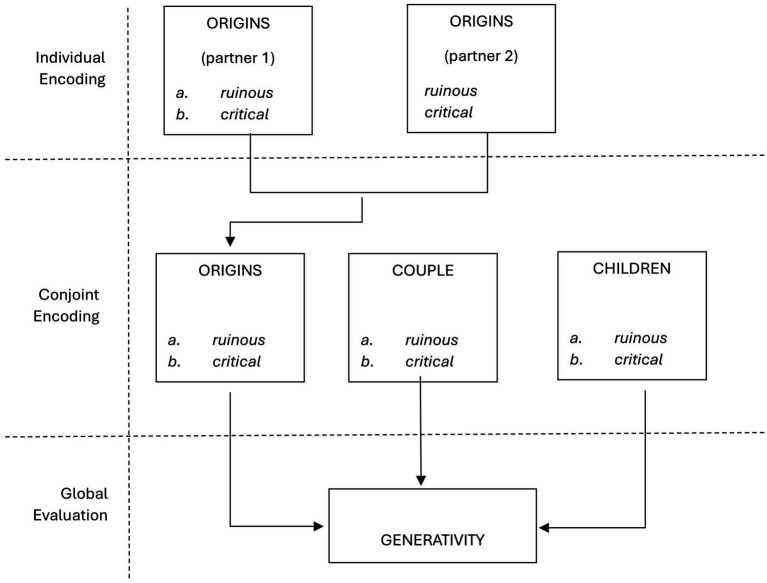
The encoding and measurement system.

On the contrary, the typological classification is based on a three-step scale (productive/critical/ruinous) and is used for the evaluation of every single item/question, as well as for the evaluation of the whole axis (origins/couple/passage) (see Figure 4).

So, it possible to analytically encode family bonds for each Interview axis (origins, couple and children) and is composed of three possible forms: productive, critical and ruinous. These forms of family bonds have been conceived and applied on a theoretical basis, starting from the reference model at the tool’s core. More specifically, productive and ruinous are, respectively, the functional and dysfunctional modes of the bond, while critical represents an uncertain bonding mode, that is to say dubious, confused, with contradictory aspects. In short, these are the three qualitatively different modes of bonds, each with its own distinctive properties.

The analytical typological encoding on the three axes is independent, in the sense that each axis of the family bond can be intrinsically traced back to each of the three forms, that is, regardless of the encoding of the other two axes. Thus, specific situations could arise, for example similar encoding on the three axes (for example an always productive bond), or different encoding on all three axes (for example a productive bond in the relationship with the origins, a critical bond in the couple relationship and a ruinous bond in the relationship with the children, even if highly improbable).

Furthermore, each form of the bond has a meaning that is specifically characterized according to the axis to which it refers. Thus, for example, the ruinous form of the bond relating to origins is qualitatively distinct from the ruinous form of the couple bond, although in both cases it is a “problematic bond,” or an indicator of de-generativity.

Lastly, a joint encoding for both partners is immediately applied to the axes relating to the couple relationship and the relationship with the children, while for the origin-related axis there is initially a specific encoding for each partner. Subsequently, on the basis of a specific combinatorial rule (see Cigoli and Tamanza, 2009), a unitary measurement is established that envisages both pure and mixed typologies.

Now we will examine beyond the labels by clarifying the meaning of each encoding category.

We will begin with origins. Productive origins are characterized by the partners’ possibility to identify themselves with members of previous generations, starting from the recognition of the resources that come from the same. This recognition involves the prevalence of feelings of trust and hope, as well as justice and fairness towards the generational bond. Although pain, grief, mistakes and shortcomings are not lacking, they are tolerated, forgiven and cleared. On the contrary, ruinous origins refer to a scorned bond in which the partners are unable to overcome the traumas endured and the suffering deriving from them. The indifference and/or abuse the partners feel victimized by lead to a scarcity of feelings which, if expressed, are negative and connoted in terms of distrust, despair and injustice. Lastly, critical origins are characterized by the presence of negative feelings which, however, do not prevent the partners from recognizing positive identifying sources and seeking the possibility of redemption.

Now we will discuss the couple bond. A productive couple bond is characterized by the partners’ ability to invest in the bond, recognizing its value and feeling their belonging to it (the “us” of a couple). A ruinous couple bond is instead characterized by each partner’s will to dominate and manipulate the other, not recognizing his or her specificity. In fact, a ruinous couple bond is an “anti-bond,” in the sense that the partners do not act in favor of the bond, but against it, even if they need it (Racamier, 1992). Among various concepts, the author has developed the concept of relational perversion. It is characterized precisely by the use-abuse of the other to achieve one’s goals. This also applies to the law, not in the sense of “that which binds men to each other,” but as a tool for achieving one’s goals. The commonality of this method is an aspect of daily life. Lastly, a critical couple bond is characterized by the presence of a constant sense of danger for themselves and for the fate of the bond, so that it is perpetually uncertain and unsettled. This does not mean that the partners do not also show aspects of intimacy and room for possible movement.

Finally, we consider the bond with the children. It is productive when an investment in the future is possible which, despite being connected and continuous with the family’s past, recognizes the children’s “proprium,” as well as recognizing the differences in the times. “Transgression” is given value precisely for this reason, in the sense of going beyond what tradition can do in order to innovate it. This can involve rituals, as well as the “rules of life” and the values that guide them.

Thus, we have two forms of transgression: one that violently attacks expectations, values and rituals and one that innovates them. This is so, for example, in the value currently attributed to “intimacy” in the bond compared to that of “respect” that has marked genders and generations. Transgression is particularly visible in its different forms in cases of family migration, where the wisdom of the older generation is frequently replaced by the technological skill and consumerist spirit of the new generation, or supported by the same skill. The parental bond is considered ruinous when a feeling of resentment and “autogenesis” prevails in the partners, which prevents the children from finding their own place within the family-generational history. Lastly, a critical bond is characterized by the presence of feelings of anguish and distrust towards the future, so that parents experience constant uncertainty both in relation to their own abilities and to their children’s destiny.

A transverse reading of the forms of bonds along the three axes suggests that each of them has constant properties beyond the axis itself. Thus, the three forms of ruinous bonds are united by the absence of movement, i.e., a very painful and “untreatable” situation that is constantly repeated. For their part, the productive forms are not as characterized by the absence of pains and limits as for the possibility of boosting the bond with trust and hope. Lastly, the critical forms show marked contradictions both on the ethical and affective sides.

The second encoding system, namely the taxonomic classification, must be referred to for the analytical classification of each axis of the family bond. This classification concerns the individual Interview stimuli, presents specific labelling categories for each of them and is associated with the corresponding “typological measurement.” Each unit of text corresponding to each stimulus of the CGI is thus read simultaneously in a qualitative manner (through the semantic categories of the taxonomy) and quantitatively (through the scale of the typology). This second measurement of the items forms the basis of the synthetic typological measurement of the axis according to an accumulative process and a prevalence criterion encoded in unique terms. At the end of the encoding process, a typological measurement of the three dimensions investigated by the CGI (origins, couple, passage) will be available alongside the semantic analysis which, through a subsequent combinatorial step, will make it possible to obtain a synthetic measurement of family generativity. The empirical work constructing and validating the CGI has made it possible to identify six different forms of generativity: fertile, evolutionary, blocked, chaotic, degenerative and poor. These are qualitatively distinct forms, each with its own peculiar characteristics and distinguished by its own space-temporality that can also be graphically depicted (see Cigoli and Tamanza, 2009, p.99).

## 6 Use in clinical assessment

The Clinical Generational Interview is a useful tool not only in research on family relationships, but also and above all in clinical practice, configuring itself as a therapeutic assessment tool. The CGI serves as a “medium” for the creation of the bond between the parental couple and the clinician, which forms a “working group” that is indispensable for good progress in the clinical pathway and its outcome. Couple assessment, in particular, has proved to be one of the elective clinical areas for the use of the CGI, since the proper setting for this tool is the joint meeting with the couple.

Alongside this homologous setting, however, there is also a substantive reason which makes it, so to speak, quite natural to imagine the use of the CGI in couples counselling. This is due to the fact that, beyond the different theoretical-conceptual references, any preliminary understanding of the couple’s functioning must, to some extent, be based on a recognition of the events and meanings that mark the foundation and development of the same couple’s story: topics that are analytically addressed by the CGI. In this regard, the specialised literature offers many conceptualisations of what function the relationship’s psychological organiser takes on; his recognisability and individuation, however, invariably comes from the reconstruction of the historical methods of the meeting and its subsequent development (see Dicks, 1967; Pincus and Dare, 1983; Ruszczyski, 1993; Losso, 2000; Zavattini, 2001). The importance that the bond with the family environments of origin and the exercise of parenting takes on is widely recognised for understanding the couple’s dynamics (Andolfi, 1988; Canevaro, 1988; Framo, 1996).

The CGI has recently been used in a systematic way in many clinical interventions with couples who were facing the same critical event, namely separation/divorce and the family’s rearrangement. These couples are those met in work contexts such as in cases requiring a Court-Appointed Counsellor and Clinical Couple’s Counselling: they are two very different and specific intervention pathways in relation to their purposes, the access modes and the institutional context, but fairly homogeneous as regards the subject and issues addressed (Gennari and Tamanza, 2017). In fact, the Court-Appointed Counsellor is arranged by the judicial authority within contentious procedures which usually feature particularly intense conflict, and has an eminently evaluative purpose. Clinical Counselling, on the other hand, starts from the independent request of the parties and has often constituted, in our case, the preliminary analysis and decoding work relating to family mediation, or couple’s psychotherapy.

From a technical point of view, both cases focus on and circumscribe pathways that primarily aim at producing an understanding of the couple’s situation and its disruptive dynamics, as well as identifying the resources that can be activated in order to achieve effective parenting and, where possible and desired, boost the relationship.

In the cases we refer to, the CGI has been used alongside other tools, including tests, but has always held a central importance because it has established the thematic track that guided the exploration of family history and the reconstruction of the couple’s story. It was initially applied in different formats to verify which administration method was best suited to the context and objectives of clinical work. After repeated attempts, we were able to verify the usefulness of introducing two variants in relation to the administrative procedure envisaged for the research activity, while still maintaining the content and formulation of the stimuli unaltered.

The first concerns the subdivision of the administration of the interview into three parts; that is, proposing the dialogic stimuli related to the three axes of the CGI in three different consecutive meetings and with a more implicit interlocutory mode. This is in relation to clinical work’s typical need of having sufficient time to retrace elements of personal and family life history in detail and in depth, which is at times marked by painful feelings or which, however, cannot often be easily recognised and shared, thus provoking resistance. Spreading out the administration time of the interview not only satisfies the requirement to accommodate people’s need to develop their narratives with appropriate times and rhythms, but first of all the need to ensure that the “working relationship” can be established as a sufficiently safe and trusting one, constituting itself as an appropriate container for the ethical-affective processes solicited by involvement in the proposed task.

The second variation concerns a different articulation of the task of choosing and commenting on the couples images. It is placed at the end of the second part of the interview and, above all, envisages that people choose - initially independently and privately - not one, but up to three images, which refer to three different temporal moments: the first refers to the present, the second refers to the past, and more precisely to the initial phase of the couple’s history and the third refers to the near future (five years later), stating that it must express how people “imagine their relationship will be,” and not as they would like it to be. The emphasis on “prediction” rather than “desire” has been much more functional in helping people confront the “factual truth” of their relational situation. A second way to use the images which has been prompted by emerging needs in clinical work with couples is to modulate the task according to the specific “critical periods” of the couple relationship. Rather than directing the choice of images according to a generic temporal succession (past-present-future), it may be useful to request the choice of an image for each significant moment (acme) of the couple relationship.

The subsequent commentary and comparison thus concern a sequence of images, facilitating and enriching reflection on the diachronic elements of the couple relationship. Not only that, the sequence of images facilitates an overall and synthetic reconstruction of the sense it assumes for each person allowing, with a relative immediacy, access to meanings and contents that often cannot be sufficiently expressed and recognised within the narrative reconstructions. As mentioned, thanks to their complex and polymorphic structure (form, content, colour, stroke, use of space, etc.), the images (the paintings) permit access to a world of meanings that articulates deep cognitive and affective contents which are more difficult to censure than the verbal language the couple can clearly control more. They therefore condense and immediately convey a multiplicity of elements that are particularly useful in order to have an overall picture of the relationship, also in reduced times.

When couples are confronting the topic (potential or current) of crisis, working with images in this way not only helps the clinician, but the subjects themselves, to reinterpret the couple’s reality and crisis in less rigid and self-centred terms (Tamanza et al., 2018). The synoptic “contemplation” of the two sequences of images manifestly demonstrates how the reality of the bond cannot be traced back to the juxtaposition of two different points of view, but refers to a complex and dynamic articulation. The comparative method of the partners’ choices in relation to the same period of the relational event makes it possible to access, in a less inferential way, the vision of the same relationship, identifying themes and elements that cross and go beyond the individual personal positions and which immediately allow access to relational rather than intra-psychological indicators. In doing so, the critical junction can be faced which concerns the need to identify methods and tools that make the scientific community’s widely developed theoretical paradigms that assign priorities to the same relationship highly operational and transmittable, understood as the true subject of clinical action compared to the individual positions of the partners (see Cigoli, 2006).

The diachronic succession of images then forces questioning the reasons and the meaning of the change (or absence of change) found in the succession itself. It also helps to examine, in more realistic terms, the existing gap between desire and reality and to search for traces and signs of a possibility to transform the relationship. In other words, the possibility of identifying resources, to be understood as tolerability of the process of overcoming the crisis, with the limits, risks and effort connected to it, as a space for movement and re-signification of what is existing in a perspective of openness to the new and to the unknown, as an assumption of responsibility for one’s own needs and desires and the world of bonds.

Lastly, the observation of the interactive and behavioral methods the couple uses to deal with the proposed task is an important source of information, both in relation to the possibility of using the resources offered in a more or less functional way within the specific counselling setting, and in a perspective and prognostic sense in relation to the possibility and usefulness of promoting subsequent intervention projects.

## 7 Final considerations

The Generational Clinical Interview, as its name explicitly indicates, is a tool for organizing the clinical encounter with the family from a psychodynamic-generational perspective. Its main intent is to constitute an aid for the investigation and evaluation of family relationships, which can combine an inclusive aspect of the complexity of the object of study with the systematicity and rigor of a structured procedure, useful for increasing the ostensibility and intersubjective validation of the knowledge it produces. A research tool that, formalized in strong coherence with the theoretical assumptions from which it derives, is not only proposed as an algorithm for testing preconstituted hypotheses, but first and foremost as a device aimed at promoting and facilitating the construction of dialogic and participatory understanding of the family relationships. The structured sequence of stimuli and the taxonomic and typological system of coding discursive productions represent the conceptual and procedural framework that guides the exploration and analysis of family ties. They also constitute a double constraint: they constrain the clinician/researcher within a dialogic-narrative canvas that is not rigid but coherent and, at the same time, they also constrain the couple in the same canvas, in a continuous guided confrontation with the origins of each of the two, with the historical and affective plot of the relationship, and with the responsibility of transmission to the children.

There are three areas of use of the Generational Clinical Interview: the first is related to research on family and couple relationships, the second is related to assessment situations, and the third is related to clinical intervention.

As much as the three areas have their specificity of “setting” (or configuration) they are also interrelated. The problem, in fact, is not so much to narrow the gap between “academic/scientific” research and field research, but to flip the relationship in favor of the clinical, remembering that without direct implication in the relational field there is no clinic, and this also applies to the researcher. In fact, the essential purpose of research is to produce the necessary information so as to be able to achieve the knowledge he or she seeks, whether it is exploratory in nature, that is, aimed at formulating descriptions and interpretative hypotheses of a given phenomenon, or evaluative in nature, aimed at corroborating or falsifying previously elaborated hypotheses. Through CGI, the researcher is directly involved in the dialogue-conversation with the couple (i.e., he or she is not external to the family relationship as in the case of the use of self-administered tests or questionnaires) and leaves room for the parental couple to *reflect* on what was experienced through the Interview. Rather, it is the very structure of CGI that, by targeting the world of relationships, creates a meaningful context from a relational perspective.

The second elective area, is that of therapeutically oriented assessment. In the context of clinical and psychosocial services, whose purpose is to structure intervention plans of various kinds and to assess the outcome of them, CGI constitutes a useful tool and procedure for relational diagnosis, that is, for assessing the generative or degenerative character of generational transitions.

Through the Interview it is indeed easy not only to focus on productive, critical, or ruinous areas of exchange, but also to mobilize some family resources from the outset and thus open an emotional and relational space for the construction of tractability.

The third set (configuration) of use of CGI is that of clinical/therapeutic intervention. It can be realized through specific and very differentiated modes of intervention, but, in any case, the Interview highlights all its value in creating a *space for sharing* and thus activating a “working group” oriented to the transformation of family relationships. Finally, since clinical psychotherapeutic work needs (and deserves) verification, the Interview, or parts of it, can serve this purpose. This leads us to a final methodological consideration: recognizing the specificities that distinguish clinical work and assuming a consequent attitude that intentionally devotes care and attention to them does not mean misrecognizing the value and necessity of using methodologically reliable tools and techniques. The fact that research and clinical intervention respond to different logics and needs does not mean that they are incompatible. Quite the contrary. Even in clinical work, in fact, it remains of essential importance to proceed systematically to the production and analysis of crucial information with respect to the object under examination (in our case precisely the world of family relationships), and for this purpose the use of structured and empirically validated tools can be particularly valuable.

In any case, the most innovative and distinctive character of CGI is the balanced synthesis between the need to proceed in a systematic and controlled manner in the collection and evaluation of crucial information related to the couple’s relationship and the need to foster a gradual and progressive active involvement of people in the clinical process. Added to this is its structurally relational orientation, that is, its ability to induce a “relational perspective,” because it forces one to think of the reality examined as a problem of relational/generational exchange and not of individuals.

Recourse to the imaginary register then produces an unexpected “displacement” with the breaking of the “escalation” mechanisms and a verbal interlocution reduced to an empty and timed script; this opens up an area for potential listening and interrogation for the couple and offers new ideas for re-defining and understanding the relationship.

The generational segmentation of the proposed topics then calls for a reconsideration of the couple’s history and its difficulties in the context of the exchange between generations, highlighting the possibility of a new definition of the parenting function in the face of the possible, or already occurred, separation. In fact, it is not uncommon for couples to move from a feeling of condemnation of themselves and/or the entire family world to pacification with their own history and with the actors who took part (understanding the conjugal relational history phase), questioning and then looking for ways to “save” the good that the relationship has produced.

However, the most relevant element is the fact that the CGI has proved to be a tool that amplifies and makes it less difficult to have a “collaborative” clinical assessment and even an expert assessment. It allows the spouses/partners to understand the meaning of their respective positions within the relational history, to clarify their respective expectations and fears and, thus, to be able to consciously choose one’s present-future. We could also say that the CGI makes it possible to move within a profoundly epistemological ethical perspective that makes sharing, participation in knowledge and responsible decision-making the main construction techniques of the clinical intervention.

## Data availability statement

The raw data supporting the conclusions of this article will be made available by the authors, without undue reservation.

## Ethics statement

Ethical approval was not required for the studies involving humans in accordance with the local legislation and institutional requirements. The participants provided their written informed consent to participate in this study.

## Author contributions

GT: Conceptualization, Data curation, Investigation, Methodology, Writing – original draft, Writing – review & editing. MG: Conceptualization, Data curation, Investigation, Methodology, Writing – original draft, Writing – review & editing.

## Footnotes

^1^The sample consisted of couples who were going through different stages of the life cycle, namely: couples with children aged 3–6 years, couples with adolescent children (14–18 years), couples with disabled children, adoptive parents and separated parents.

^2^Following Clark and Watson (1995), a frequency distribution analysis was conducted on the initial pool of items (in order to exclude those items with high rates of nondeterminability or excessive concentration in a single response mode) and then a confirmatory factor analysis (CFA) (Stewens, 1996) was conducted.

^3^In fact, the preliminary version included 35 dialogic-discursive stimuli, while this second version consists of 23 items.

^4^This second sample consisted of sixty pairs, thirty of which were nonclinical (and divided into different stages of the life cycle, like the first sample) and thirty marked by specific clinical and social issues.

^5^Linguistic-textual analysis was carried out with the help of T-LAB, a software program that, by means of numerous algorithms, allows for a series of in-depth text operations of an exploratory and interpretative nature. (Lancia, 2004).
