# Supplementary material for: The Clinical Generational Interview. An instrument for family assessment
Source: Front Psychol. 2024 May 31;15:1361028. doi: 10.3389/fpsyg.2024.1361028 (PMC11177784; doi:10.3389/fpsyg.2024.1361028)
Supplement: Supplementary file 1 [file Data_Sheet_1.docx]

**Appendix 1 GCI Encoding and calculation sheet**

| ***FIRST AXIS: ORIGINS*** | | | | | | | |  |
| --- | --- | --- | --- | --- | --- | --- | --- | --- |
| **Item** | | **Taxonomic classification** | **Typological classification** | | **MT coding** | | **MG Coding** |  |
| A1 | Environment of origin |  | 0 Undefinable | |  | |  |  |
|  |  | Poor mentalisation | 1 Ruinous | |  | |  |  |
|  |  | Doubtful, contradictory feelings | 2 Critical | |  | |  |  |
|  |  | Positive or ambivalent feelings | 3 Productive | |  | |  |  |
| A2 | Comment on the image |  | 0 Undefinable | |  | |  |  |
|  | chosen | Poor comment | 1 Ruinous | |  | |  |  |
|  |  | Uncertain or negative feelings | 2 Critical | |  | |  |  |
|  |  | Mostly positive feelings | 3 Productive | |  | |  |  |
| A3 | The "golden rules" |  | 0 Undefinable | |  | |  |  |
|  |  | Lack of rules | 1 Ruinous | |  | |  |  |
|  |  | Rigid and oppressive rules | 1 Ruinous | |  | |  |  |
|  |  | Formal rules | 2 Critical | |  | |  |  |
|  |  | Participatory rules | 3 Productive | |  | |  |  |
|  |  |  |  | |  | |  |  |
| A4 | Relationship with mother |  | 0 Undefinable | |  | |  |  |
|  |  | Deficient, misleading | 1 Ruinous | |  | |  |  |
|  |  | Doubtful, contradictory | 2 Critical | |  | |  |  |
|  |  | Constructive, expansive | 3 Productive | |  | |  |  |
| A5 | Relationship with father |  | 0 Undefinable | |  | |  |  |
|  |  | Deficient, misleading | 1 Ruinous | |  | |  |  |
|  |  | Doubtful, contradictory | 2 Critical | |  | |  |  |
|  |  | Constructive, expansive | 3 Productive | |  | |  |  |
| A6 | Relationship between siblings |  | 0 Undefinable | |  | |  |  |
|  |  | Deficient, misleading | 1 Ruinous | |  | |  |  |
|  |  | Doubtful, contradictory | 2 Critical | |  | |  |  |
|  |  | Constructive, expansive | 3 Productive | |  | |  |  |
| A7 | Couple |  | 0 Undefinable | |  | |  |  |
|  | relationship learning | Devaluation, division | 1 Ruinous | |  | |  |  |
|  |  | Idealisation, repetition | 2 Critical | |  | |  |  |
|  |  | Valorisation, transformation | 3 Productive | |  | |  |  |
| A8 | Relationship with lineages |  | 0 Undefinable | |  | |  |  |
|  |  | Removal | 1 Ruinous | |  | |  |  |
|  |  | Very painful | 1 Ruinous | |  | |  |  |
|  |  | Doubtful, contradictory | 2 Critical | |  | |  |  |
|  |  | Constructive, expansive bonds | 3 Productive | |  | |  |  |
| **Origins Axis Encoding** | | | |  | |  | | |

| ***SECOND AXIS: COUPLE*** | | | | |
| --- | --- | --- | --- | --- |
| **Item** | | **Taxonomic classification** | **Typological classification** | **Encoding** |
| B1 | The meeting |  | 0 Undefinable |  |
|  |  | For need, necessity | 1 Ruinous |  |
|  |  | By chance | 2 Critical |  |
|  |  | It was a favourable time | 3 Productive |  |
| B2 | From meeting to bond |  | 0 Undefinable |  |
|  |  | There is no distinction | 1 Ruinous |  |
|  |  | Deficient couple identity | 2 Critical |  |
|  |  | Search for a couple identity | 3 Productive |  |
| B3 | What have you married... |  | 0 Undefinable |  |
|  |  | No recognition | 1 Ruinous |  |
|  |  | Partial recognition | 2 Critical |  |
|  |  | Recognition of needs | 3 Productive |  |
| B4 | Have you found what |  | 0 Undefinable |  |
|  | you were looking for... | No | 1 Ruinous |  |
|  |  | Partly | 2 Critical |  |
|  |  | Yes | 3 Productive |  |
| B5 | New discoveries… |  | 0 Undefinable |  |
|  |  | New demeaning aspects | 1 Ruinous |  |
|  |  | Feeling of stagnation | 1 Ruinous |  |
|  |  | New aspects that put couple at risk | 2 Critical |  |
|  |  | New aspects that enliven the couple | 3 Productive |  |
| B6 | Difficult moments... |  | 0 Undefinable |  |
|  |  | Impossibility to face the crisis | 1 Ruinous |  |
|  |  | Relevant difficulties with serious risk | 2 Critical |  |
|  |  | Difficulties faced | 3 Productive |  |
| B7 | Comment on the images |  | 0 Undefinable |  |
|  | chosen | Division of worlds | 1 Ruinous |  |
|  |  | Poor dialogue, recognised difficulty | 2 Critical |  |
|  |  | Active dialogue | 3 Productive |  |
| B8 | Meeting with the family |  | 0 Undefinable |  |
|  | of origin of the other... | Refusal | 1 Ruinous |  |
|  |  | Detached | 1 Ruinous |  |
|  |  | Unbalanced | 2 Critical |  |
|  |  | Reciprocal | 3 Productive |  |
| B9 | The couple's future... |  | 0 Undefinable |  |
|  |  | Negative, anguished... | 1 Ruinous |  |
|  |  | Doubtful, fearful | 2 Critical |  |
|  |  | Positive, investment and hope | 3 Productive |  |
|  | **Couple Axis Encoding** | | |  |

| ***THIRD AXIS: PASSAGE*** | | | | | | | | |
| --- | --- | --- | --- | --- | --- | --- | --- | --- |
| **Item** | | | **Taxonomic classification** | | **Typological classification** | | **Encoding** | |
|  | |  |  | |  | |  | |
| C1 | Prefiguration of | | |  | | 0 Undefinable | |  |
|  | family life... | | | Absence of prefigurations | | 1 Ruinous | |  |
|  |  | | | Stereotypical images | | 1 Ruinous | |  |
|  |  | | | Divergent perspectives, doubts, fears | | 2 Critical | |  |
|  |  | | | Trust, hope, pleasure | | 3 Productive | |  |
| C2 | Which expectations have been fulfilled | | |  | | 0 Undefinable | |  |
|  | and which not... | | | Flattening, automatism | | 1 Ruinous | |  |
|  |  | | | Intolerable/traumatic gap | | 1 Ruinous | |  |
|  |  | | | Divergences, doubts, fears | | 2 Critical | |  |
|  |  | | | Tolerable and addressed gap | | 2 Critical | |  |
|  |  | | | Constructive and enlivening gap | | 3 Productive | |  |
| C3 | Values ​​and models of the | | |  | | 0 Undefinable | |  |
|  | passage to children... | | | Absence of values, stereotyping | | 1 Ruinous | |  |
|  |  | | | Doubtful values | | 2 Critical | |  |
|  |  | | | Identification of precise values | | 3 Productive | |  |
| C4 | Sense of parental | | |  | | 0 Undefinable | |  |
|  | effectiveness... | | | Feeling of ineffectiveness | | 1 Ruinous | |  |
|  |  | | | Doubtful feeling | | 2 Critical | |  |
|  |  | | | Constructive, functional feeling | | 3 Productive | |  |
| C5 | Specific identification | | |  | | 0 Undefinable | |  |
|  | of the children... | | | Replication, duplication | | 1 Ruinous | |  |
|  |  | | | Doubt, confusion | | 2 Critical | |  |
|  |  | | | Multiple and specific belonging | | 3 Productive | |  |
| C6 | Pain and hope/trust | | |  | | 0 Undefinable | |  |
|  | in family life... | | | Anger, anguish, failure | | 1 Ruinous | |  |
|  |  | | | Doubt and confusion | | 2 Critical | |  |
|  |  | | | Faith and hope | | 3 Productive | |  |
| **Passage Axis Encoding** | | | | | | |  | |
